# Epidermal hepcidin is required for neutrophil response to bacterial infection

**DOI:** 10.1172/JCI126645

**Published:** 2019-12-03

**Authors:** Mariangela Malerba, Sabine Louis, Sylvain Cuvellier, Srikanth Mairpady Shambat, Camille Hua, Camille Gomart, Agnès Fouet, Nicolas Ortonne, Jean-Winoc Decousser, Annelies S. Zinkernagel, Jacques R.R. Mathieu, Carole Peyssonnaux

**Affiliations:** 1 Université de Paris, Institut Cochin, INSERM, CNRS, F-75014 Paris, France.; 2 Laboratory of Excellence GR-Ex, Paris, France.; 3 Department of Infectious Diseases and Hospital Epidemiology, University Hospital Zurich, University of Zurich, Zurich, Switzerland.; 4 Service de Dermatologie, Assistance Publique-Hôpitaux de Paris, Paris, France.; 5 Hôpital Henri Mondor, Assistance Publique-Hôpitaux de Paris, Créteil, France.; 6 EA 7379 EPiderME, Université Paris Est Créteil, Créteil, France.; 7 Laboratoire de Bactériologie Hygiène and; 8 Equipe Opérationnelle d’Hygiène, Assistance Publique-Hôpitaux de Paris, Paris, France.; 9 EA 7380 Dynamyc, Université Paris-Est Créteil, Créteil, France.; 10 Ecole Nationale Vétérinaire d’Alfort (EnvA), Maisons-Alfort, France.; 11 Faculté de Médecine de Créteil, Université Paris Est Créteil, Créteil, France.; 12 Pathology Department, Henri Mondor Hospital, Assistance Publique-Hôpitaux de Paris, Créteil, France.

**Keywords:** Infectious disease, Bacterial infections, Chemokines, Mouse models

## Abstract

Novel approaches for adjunctive therapy are urgently needed for complicated infections and patients with compromised immunity. Necrotizing fasciitis (NF) is a destructive skin and soft tissue infection. Despite treatment with systemic antibiotics and radical debridement of necrotic tissue, lethality remains high. The key iron regulatory hormone hepcidin was originally identified as a cationic antimicrobial peptide (AMP), but its putative expression and role in the skin, a major site of AMP production, have never been investigated. We report here that hepcidin production is induced in the skin of patients with group A *Streptococcus* (GAS) NF. In a GAS-induced NF model, mice lacking hepcidin in keratinocytes failed to restrict systemic spread of infection from an initial tissue focus. Unexpectedly, this effect was due to its ability to promote production of the CXCL1 chemokine by keratinocytes, resulting in neutrophil recruitment. Unlike CXCL1, hepcidin is resistant to degradation by major GAS proteases and could therefore serve as a reservoir to maintain steady-state levels of CXCL1 in infected tissue. Finally, injection of synthetic hepcidin at the site of infection can limit or completely prevent systemic spread of GAS infection, suggesting that hepcidin agonists could have a therapeutic role in NF.

## Graphical abstract

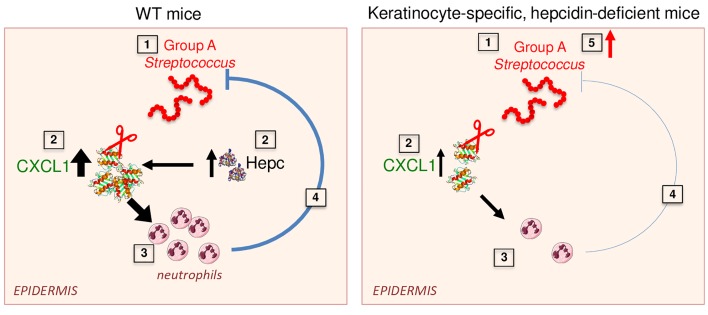

## Introduction

Necrotizing fasciitis (NF) is an infection characterized by widespread necrosis of the skin, subcutaneous tissues, and fascia that was first described by Hippocrates in the 5th century (1). The standard treatment of NF consists of broad-spectrum antibiotics, extensive surgical debridement, and supportive care. However, even with current state-of-the-art treatment, NF frequently takes a fulminant course and is still associated with high mortality rates up to 35% (1). Group A *Streptococcus* (GAS) is considered the most common cause of NF associated with bacteremia and shock. Upon detection of these Gram-positive pyogenic bacteria, neutrophil recruitment is critical to the resolution of infection (2). However, GAS is equipped with a magnitude of virulence factors, allowing the pathogen to uniquely counteract each antibacterial strategy of neutrophils (3).

Hepcidin was originally identified as a cationic antimicrobial peptide (AMP) by its close structural similarity to the beta defensins but is now also recognized as a key iron regulatory hormone (4). Hepcidin is mainly produced by the liver in conditions of high iron, infection, or inflammation. Hepcidin controls plasma iron levels by binding to ferroportin (FPN), the only known iron exporter, and inducing its degradation (5). Patients with iron overload are well known to be associated with a predisposition to a variety of infections. Hepcidin contributes to innate immunity by decreasing plasma iron levels, providing an iron-restricted internal milieu inhospitable to microbes (6).

Besides the liver, an increasing number of studies showed that hepcidin is also expressed in other tissues (7–10). We previously demonstrated that hepatic hepcidin is sufficient to ensure systemic iron homeostasis in physiological conditions (11), suggesting that production of hepcidin by other tissues may have local roles. It may have a role at the site of infections and/or in poorly perfused tissues, inaccessible by systemic hepcidin from the circulation. The putative expression and local role of hepcidin in the skin, a major site of AMP production, are not known. We have employed our recently generated mouse model, in which the hepcidin gene can be spatiotemporally inactivated, to explore the putative expression and role of hepcidin in the skin in the context of GAS infection.

## Results and Discussion

**Figure 1:**
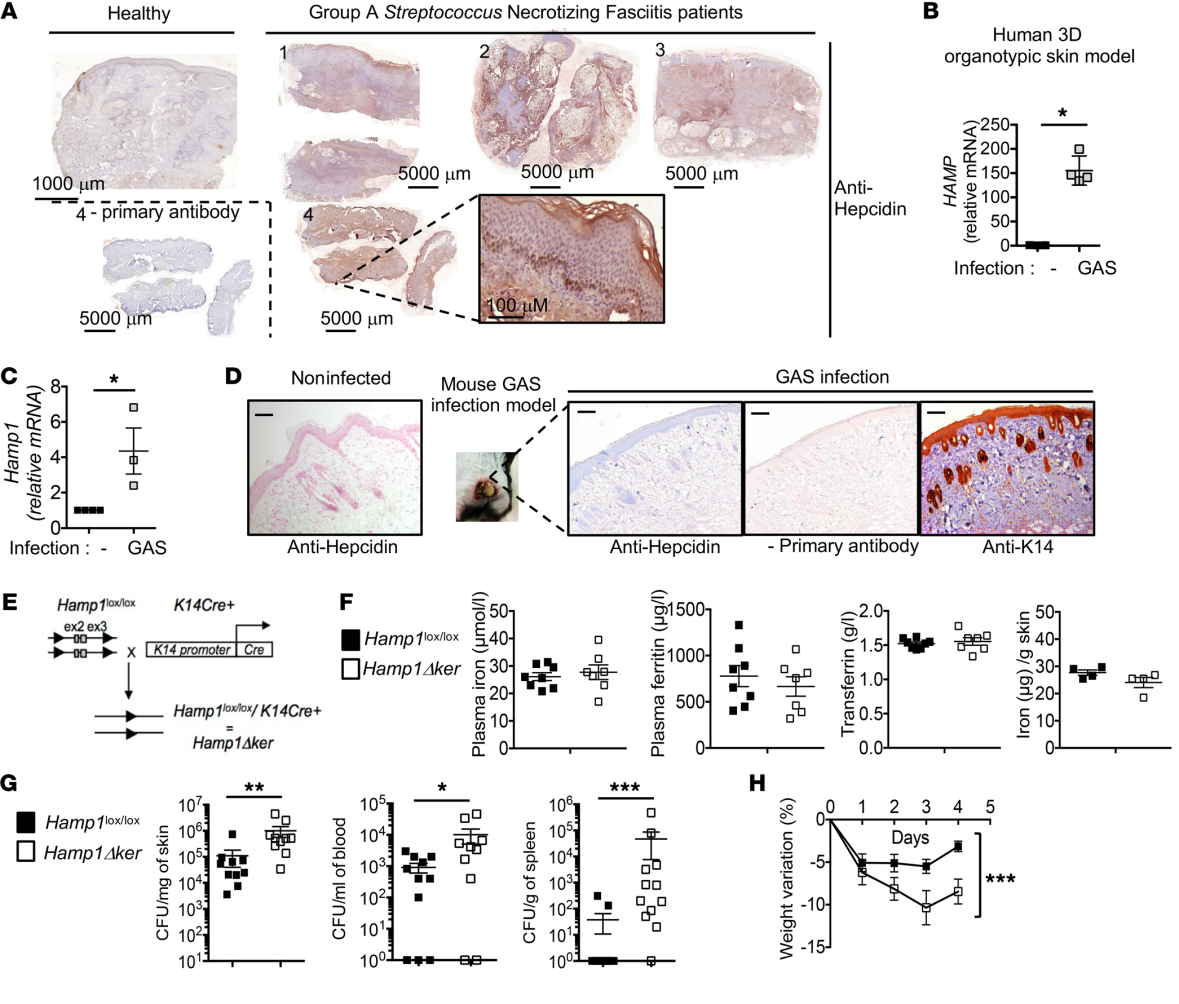
Keratinocyte hepcidin prevents bacterial systemic spread. IHC with or without primary antibody detecting (**A**) hepcidin (in brown) on sections of cutaneous human biopsies of GAS NF patients and healthy control using PerkinElmer’s Lamina multilabel slide scanner Panoramic Viewer software. (**B**) Real-time reverse transcription PCR (qPCR) for hepcidin from GAS-infected human 3D organotypic skin equivalent model; *n* = 4 per group. (**C**) qPCR for hepcidin in murine GAS-infected skin; *n* ≥ 3 per group. (**D**) Hepcidin (in blue) and K14 (in brown) IHC on cutaneous biopsies of WT mice challenged or not with GAS. Scale bars: 100 μm. Leica DMI3000B microscope, Leica DFC310FX camera, 5/0.4; Leica LAS Core software. (**E**) Generation of *Hamp1*Δ^ker^ mice. (**F**) Plasma iron, ferritin, transferrin, and skin iron levels in *Hamp1*^lox/lox^ and *Hamp1*Δ^ker^ mice; *n* ≥ 4 per group. (**G**) Bacterial count in skin, blood, and spleen of *Hamp1*^lox/lox^ and *Hamp1*Δ^ker^ mice 4 days after injection with GAS; *n* ≥ 10 per group. (**H**) Weight variation of *Hamp1*^lox/lox^ and *Hamp1*Δ^ker^ mice during infection; *n* = 10 per group. Statistical analysis was performed using a Mann Whitney test (**B**, **C**, **F**, and **G**) or a 2-way ANOVA followed by Tukey’s test for weight kinetics (**H**). *P < 0.05; **P < 0.01; ***P < 0.001.

We examined hepcidin expression on skin biopsies derived from patients suffering from GAS NF (detailed in Supplemental Table 1; supplemental material available online with this article; https://doi.org/10.1172/JCI126645DS1). Hepcidin staining of human liver tissue sections was used as a positive control (Supplemental Figure 1). Hepcidin expression was higher and more widespread in the skin of NF patients than in the skin of a healthy subject, especially in keratinocytes, the predominant cell type in the epidermis (Figure 1A). Hepcidin mRNA expression was induced (Figure 1B) in a human 3D organotypic skin model (Supplemental Figure 2) as a direct consequence of GAS infection. To investigate the role of hepcidin in the development of NF, we used an established model of necrotizing soft tissue infection (12, 13) where a strain of GAS, isolated from a patient with NF (14), is introduced subcutaneously into a shaved area on the flank of a mouse. Compared with skin biopsies of healthy mice, hepcidin expression was induced in the skin of infected mice (Figure 1C) and clearly detected in the keratinocytes, as visualized by keratin 14 (K14) staining (Figure 1D).

To probe the functional significance of keratinocyte-derived hepcidin in vivo, we developed a mouse model of keratinocyte-specific hepcidin deficiency (*Hamp1*Δ^ker^) by crossing *Hamp1*^lox/lox^ mice with K14^cre+^ mice (Figure 1E). We observed an efficient truncation of the floxed *Hamp1* allele in the epidermis of the *Hamp1*Δ^ker^ mice, but not in the *Hamp1*^lox/lox^ mice or K14^cre+^ mice (Supplemental Figure 3; see complete unedited blots in the supplemental material). Systemic iron parameters were unchanged between *Hamp1*^lox/lox^ and *Hamp1*Δ^ker^ mice (Figure 1F), in agreement with our previous study (11), suggesting that hepcidin production by extrahepatic tissues does not contribute to systemic iron homeostasis. Iron levels were also similar in the skin of *Hamp1*Δ^ker^ and *Hamp1*^lox/lox^ mice (Figure 1F).

These mice were infected with GAS in the NF model (14). Keratinocyte hepcidin staining was not detectable in the *Hamp1*Δ^ker^ mice (Supplemental Figure 4), confirming that the stained hepcidin peptide is of skin but not of liver origin. Four days after infection, *Hamp1*Δ^ker^ mice had a significantly higher number of bacteria than the *Hamp1*^lox/lox^ littermates at the lesion site (10^6^ vs 10^5^ CFU/mg) but also in the blood (10^4^ vs 9 × 10^2^ CFU/mL) and in the spleen (5 × 10^4^ vs 38 CFU/g) (Figure 1G). *Hamp1*Δ^ker^ mice also lost more weight than the *Hamp1*^lox/lox^ mice, further underlining the higher morbidity in these mice (Figure 1H). These data indicate that keratinocyte production of hepcidin is important in limiting the ability of GAS to replicate within the necrotic skin tissues and to disseminate from the initial focus of infection into the bloodstream and systemic organs.

**Figure 2:**
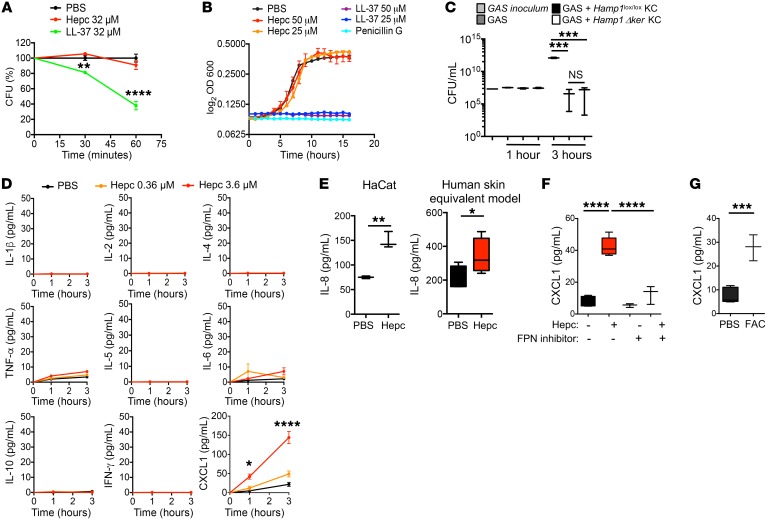
Hepcidin promotes CXCL1 production by keratinocytes. (**A**) GAS killing kinetics with 32 μM of LL-37 and hepcidin; *n* = 3 per group. Representative of 2 independent experiments. (**B**) GAS growth curve in the presence of penicillin G, LL-37, hepcidin, or PBS; *n* = 3 per group. Representative of 2 independent experiments. (**C**) Bacterial recovering at 1 hour and 3 hours following incubation of log-phase GAS with murine primary keratinocytes (KC) from *Hamp1*^lox/lox^ and *Hamp1*Δ^ker^ mice. Data are representative of 2 independent experiments performed in triplicate. (**D**) Cytokines measured with the V-PLEX Proinflammatory Panel1 kit in the culture supernatant of murine primary keratinocytes stimulated for 1 or 3 hours with hepcidin or PBS; *n* = 3 per group. Representative of 3 independent experiments. (**E**) IL-8 ELISA on the culture supernatant of HaCat or a human 3D skin equivalent model stimulated with 3.6 μM hepcidin; *n* ≥ 3 per group. (**F**) CXCL1 levels measured by ELISA in the culture supernatant of murine primary keratinocytes stimulated for 3 hours with 3.6 μM hepcidin in the presence of PBS or 100 μM FPN inhibitor (2D-014); *n* ≥ 3 per group. Representative of 3 independent experiments. (**G**) CXCL1 levels measured by ELISA on the culture supernatant of murine primary keratinocytes stimulated for 3 hours with 500 μM ferric ammonium citrate (FAC); *n* = 3 per group. Representative of 3 independent experiments. Statistical analysis was performed using a 2-way ANOVA followed by Tukey’s test (**A**, **B**, and **D**), unpaired Student’s *t* test (**E** and **G**), or a 1-way ANOVA followed by Tukey’s test (**C** and **F**). *P < 0.05; **P < 0.01; ***P < 0.001; ****P < 0.0001.

To investigate the mechanisms by which hepcidin protected against the spread of GAS infection, we first determined the putative bacteriostatic and bactericidal effects of hepcidin against GAS in vitro. While the well-known antimicrobial peptide LL-37 demonstrated bacteriostatic activities against GAS (Figure 2A), hepcidin had neither bactericidal (Figure 2A) nor bacteriostatic activities (Figure 2B). Moreover, primary keratinocytes derived from *Hamp1*^lox/lox^ and *Hamp1*Δ^ker^ mice displayed the same bactericidal activity against this pathogen (Figure 2C). We therefore ruled out a direct antimicrobial effect of hepcidin on these bacteria.

AMPs have been reported to have pleiotropic effects and influence a host’s inflammatory responses during infection (15). We therefore asked whether hepcidin could have an immunomodulatory role in keratinocytes. For this purpose, we performed a cytoplex on the supernatant of murine primary keratinocytes incubated with 0.36 μM and 3.6 μM synthetic hepcidin. Interestingly, hepcidin induced a dose-dependent increase of the key neutrophil chemokine CXCL1 but not of the other inflammatory cytokines we tested (Figure 2D). The capacity of mouse hepcidin to induce CXCL1 in primary keratinocytes was confirmed by ELISA (Supplemental Figure 5), as was the capacity of human hepcidin to induce the production of IL-8, the human functional homolog of CXCL1, in the human HaCat keratinocyte cell line and in a human 3D organotypic skin model (Figure 2E).

The cognate receptor of hepcidin is the iron exporter FPN, questioning the role of FPN/iron in the induction of CXCL1 by hepcidin. The stimulatory effect of hepcidin on CXCL1 was reduced by the addition of a drug preventing the interaction of hepcidin with the iron exporter FPN (16) (Figure 2F). These data suggest that hepcidin, in primary keratinocytes, induces CXCL1 through a FPN-dependent pathway. Binding of hepcidin to FPN is well known to induce its internalization and degradation, resulting in an increase of intracellular iron (5). In corroboration with the action of hepcidin on FPN, incubation of primary keratinocytes with iron stimulated CXCL1 production (Figure 2G).

**Figure 3:**
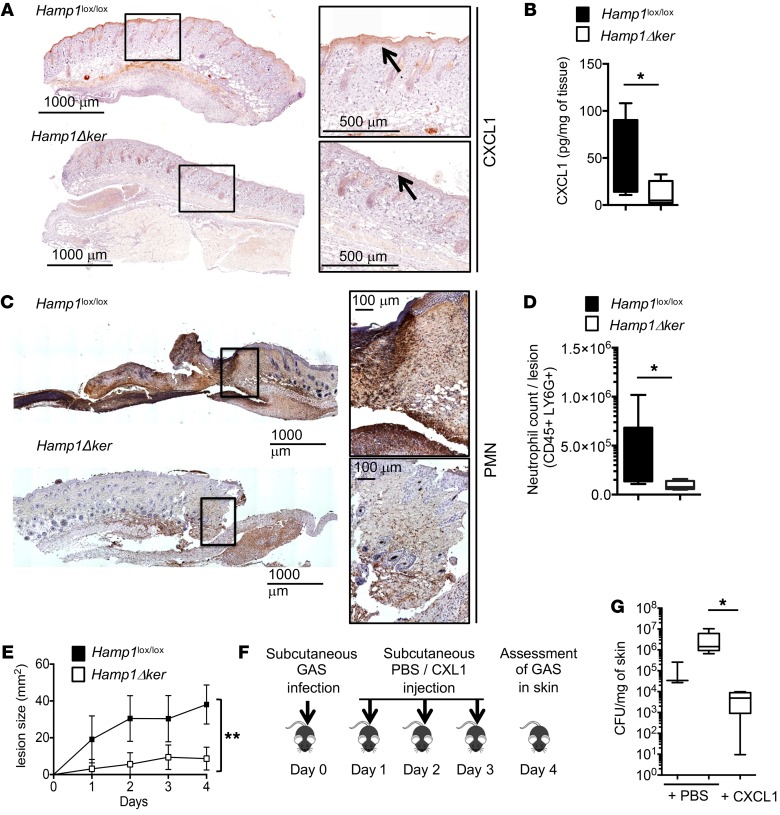
Hepcidin is required for CXCL1 production and neutrophil recruitment. (**A**) Anti-CXCL1 or (**C**) anti–polymorphonuclear leucocyte (PMN) immunostainings on skin of *Hamp1*^lox/lox^ and *Hamp1*Δ^ker^ mice challenged with GAS. PerkinElmer’s Lamina multilabel slide scanner Panoramic Viewer software. (**B**) CXCL1 ELISA on lysates from GAS-infected skin biopsies of *Hamp1*^lox/lox^ (*n* = 5) and *Hamp1*Δ^ker^ mice (*n* = 6). (**D**) Neutrophil count from GAS-infected skin biopsies of *Hamp1*^lox/lox^ (*n* = 5) and *Hamp1*Δ^ker^ mice (*n* = 4). (**E**) Area of necrotic ulcers in skin of *Hamp1*^lox/lox^ and *Hamp1*Δ^ker^ mice during GAS infection; *n* = 7 per group. (**F**) Scheme of the study protocol. (**G**) Bacterial count in the skin of *Hamp1*^lox/lox^ and *Hamp1*Δ^ker^ mice injected daily with CXCL1 or PBS; *n* ≥ 4 per group. Statistical analysis was performed using a Student’s *t* test (**B** and **D**), a 2-way ANOVA followed by Tukey’s test (**E**), or a 1-way ANOVA followed by Tukey’s test (**G**). *P < 0.05; **P < 0.01.

In agreement with the in vitro results showing that hepcidin stimulates CXCL1 production in primary keratinocytes, the in vivo keratinocyte CXCL1 production in response to GAS infection was lower in *Hamp1*Δ^ker^ mice than in *Hamp1*^lox/lox^ littermates, as shown by IHC (Figure 3A) and ELISA (Figure 3B) on skin biopsies. As a consequence of the lower CXCL1 production, less neutrophil recruitment was observed in the skin of *Hamp1*Δ^ker^ mice compared with that of control littermates, as shown by IHC (Figure 3C) and by cytometry analysis (Figure 3D). This defect in the ability of keratinocyte-derived hepcidin to recruit neutrophils at the site of infection translated into a decrease in the necrotic skin lesion size of the *Hamp1*Δ^ker^ mice as compared with controls (Figure 3E). Subcutaneous injection of CXCL1 into GAS-infected *Hamp1*Δ^ker^ mice (Figure 3F) significantly decreased the number of bacteria to even below that found in the lesions of *Hamp1*^lox/lox^ mice (Figure 3G). These results strongly suggest that the lack of CXCL1 production in *Hamp1*Δ^ker^ mice was responsible for their susceptibility to GAS infection. Altogether, these results suggest that hepcidin is critical for regulating CXCL1 production in keratinocytes and that it may tune the magnitude of the neutrophil recruitment in the immune response.

**Figure 4:**
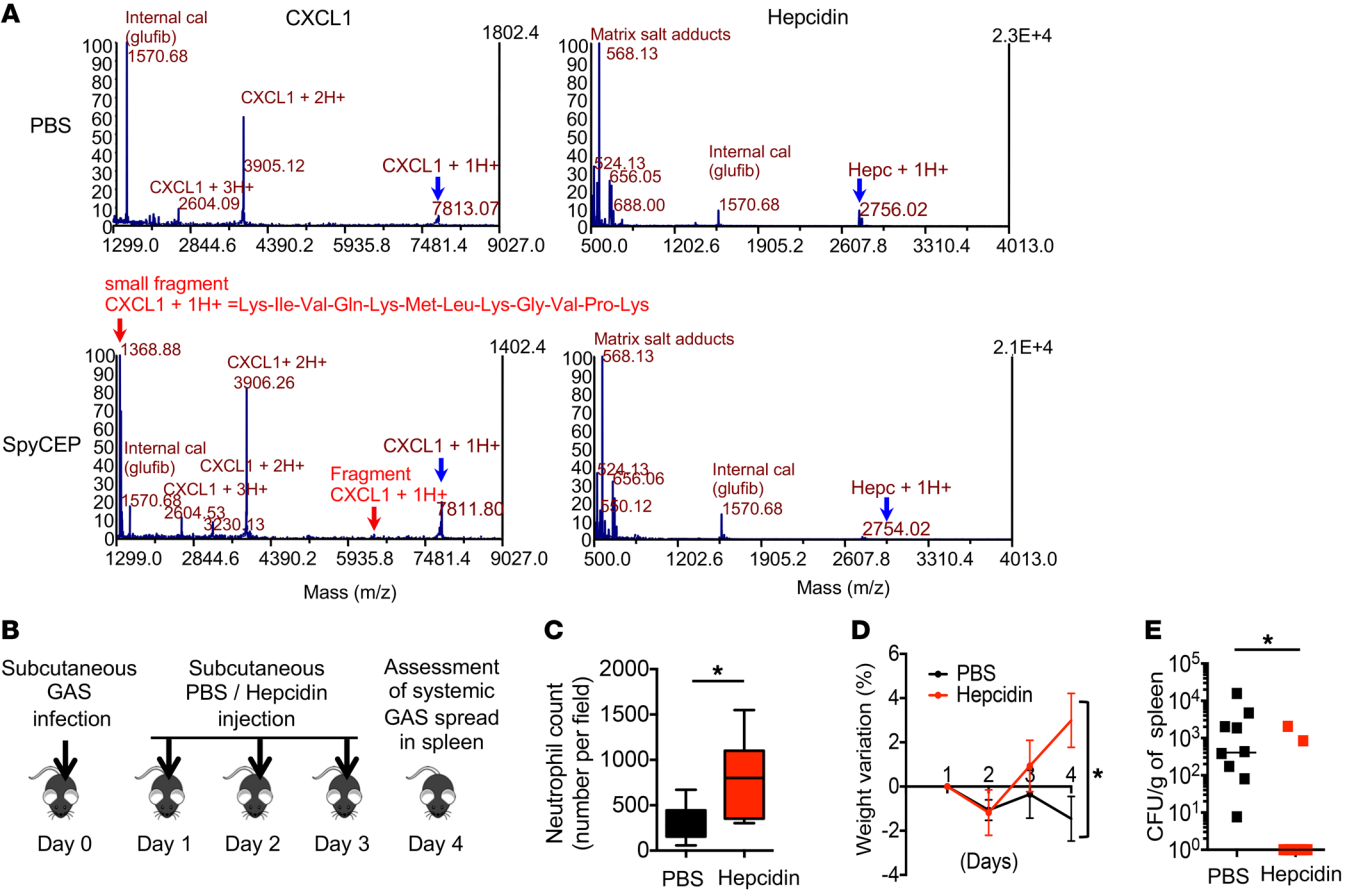
Hepcidin is resistant to SpyCEP cleavage and has a therapeutic role in NF. (**A**) Mass spectroscopy analysis of CXCL1 or hepcidin incubated overnight with SpyCEP or PBS. Electrospray ionization generated a series of multiply charged ions (indicated as m/z; mass-to-charge ratio) from which the average molecular mass (m) of each was deduced. The blue arrows indicate uncleaved peptide peaks at 7.8 kDa (CXCL1) and 2.7 kDa (hepcidin). Red arrows show the cleavage products of CXCL1 with a small (1.3 kDa) and a big (5.9 kDa) fragment. (**B**) Therapeutic protocol. (**C**) Neutrophil count (3 measurements per individual mouse were averaged); *n* = 6 per group. (**D**) Weight variation and (**E**) bacterial count in spleen of WT infected mice treated with PBS or hepcidin (*n* = 9, red square) or PBS (*n* = 9, black square) during 4 days. Statistical analysis was performed using a Student’s *t* test (**C**), a 2-way ANOVA followed by Tukey’s test (**D**), or a Mann Whitney test (**E**). *P < 0.05.

We next investigated the possible advantages of indirect production of CXCL1 through hepcidin during GAS infection. GAS is equipped with a quantity of neutrophil resistance factors, allowing the pathogen to uniquely counteract each antibacterial strategy of neutrophils (3). One of the principal mechanisms of GAS-immune escape is the production of surface-associated serine proteases by GAS, such as SpyCEP (also designated ScpC), which cleaves human IL-8/mouse CXCL1 to suppress chemokine-mediated neutrophil recruitment (17), or SpeB, which allows GAS to translocate across the epithelial barrier by degrading several host plasma and matrix proteins (18). To examine whether hepcidin was a target of SpyCEP or SpeB, synthetic hepcidin and CXCL1 (as a control) were incubated with purified SpyCEP, SpeB, or PBS overnight. The digestion products were examined by mass spectrometry analyses. As shown by the MALDI spectrum (Figure 4A), CXCL1 was cleaved, as expected, into 2 fragments of 5.9 kDa and 1.3 kDa. In contrast, hepcidin was not cleaved in the presence of SpyCEP (Figure 4A, bottom panel) or SpeB (Supplemental Figure 6). This suggests that hepcidin could serve as a reservoir to maintain a steady-state level of CXCL1 in the context of infection. CXCL1 is phylogenetically ancient and is expressed in *Dictostelium discoideum,* whereas hepcidin-like peptides appeared more recently during evolution, for example in teleost fishes (Supplemental Figure 7). Considering the theory that host-pathogen interactions coevolve, we could speculate that whereas GAS has already evolved to counteract the activity of CXCL1, it has not yet developed a virulence factor able to neutralize the activity of hepcidin. Because of hepcidin resistance to bacterial protease activities such as SpyCEP or SpeB, and in view of its unanticipated immunomodulatory role, we also asked whether local hepcidin injection could have a therapeutic effect on the systemic spread of bacteria in a NF model. Twenty-four hours after GAS infection, 1 μg synthetic hepcidin or PBS was subcutaneously injected at the bacterial inoculation site, followed by 2 injections of 500 ng hepcidin or PBS for 2 consecutive days (Figure 4B). As expected, hepcidin-treated mice showed an increase in neutrophil recruitment (Figure 4C). In contrast to the PBS-treated mice, which exhibited systemic signs of infection including weight loss (Figure 4D), rough hair coat, and hunched posture (data not shown), hepcidin-treated mice did not present any signs of systemic disease and accordingly recovered their initial weight. Remarkably, whereas all the control mice presented with systemic bacterial dissemination (as shown by the number of bacteria in the spleen), 7 of the 9 hepcidin-treated mice showed absolutely no bacterial dissemination (Figure 4E). Hepcidin treatment did not prevent bacterial dissemination in CXCL1^–/–^ mice (Supplemental Figure 8), showing that the therapeutic effect of hepcidin acted through CXCL1. Hepcidin thus demonstrated a therapeutic role in this mouse model of NF. These results suggest that hepcidin represents an alternative additional strategy for treating GAS-derived NF, and that it merits further investigation, especially in view of the increasing incidence of invasive GAS disease worldwide (19).

Altogether, we could speculate that skin hepcidin status (low vs high) may be a marker of sepsis development. Future studies should investigate whether skin hepcidin levels inversely correlate with the severity of sepsis development in patients with NF.

In addition to its key role as an iron regulatory hormone produced by the liver, we have demonstrated here that epidermal hepcidin may also be an essential heretofore unrecognized component of the immune response to bacterial infection. Modulation of its expression may represent a novel therapeutic approach for patients with necrotizing fasciitis.

## Methods

See Supplemental Methods.

### Study approval.

For human studies, written informed consent to the protocol was obtained for all subjects and was approved by the Institutional Review Board and the regional ethics committee Paris IV (IRB 2016/40NICB and IRB 00003835). The collection of personal data was approved by the “Commission Nationale de l’Informatique et des Libertés.”

The animal studies described here were reviewed and approved (agreement no. CEEA34.CP.003.13) by the “Président du Comité d’Ethique pour l’Expérimentation Animale Paris Descartes” and are in accordance with the principles and guidelines established by the European Convention for the Protection of Laboratory Animals (Council of Europe, ETS 123, 1991).

## Author contributions

MM, SL, SC, SMS, and JRRM designed the experiments, carried out experiments, and performed data analysis. AF, ASZ, CH, CG, NO, and JWD provided essential reagents and scientific advice. JRRM and CP designed the experiments and supervised the project. CP wrote the manuscript.

## Supplementary Material

Supplemental data

## Version 1 10/10/2019

In-Press Preview

## Version 2 12/03/2019

Electronic publication

## Version 3 01/02/2020

Print issue publication
