# Supplementary material for: Epidermal hepcidin is required for neutrophil response to bacterial infection
Source: J Clin Invest. 2019 Dec 3;130(1):329–34. doi: 10.1172/JCI126645 (PMC6934188; doi:10.1172/JCI126645)

SUPPLEMENTARY INFORMATION

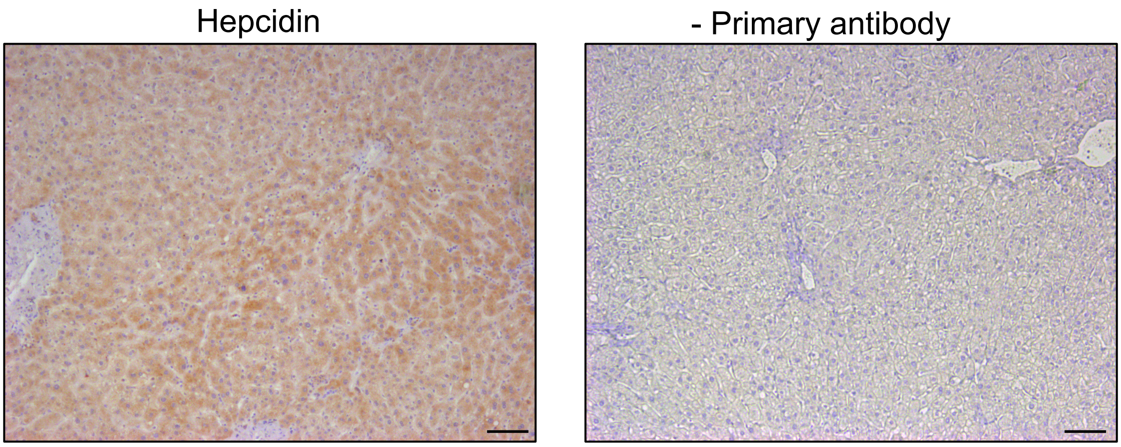

Supplementary Figure 1. Immunohistochemistry with or without primary antibody detecting hepcidin on sections of liver human biospies of an heathy subject. Bars represent 100 μm. (10/0.4, Leica DMI3000B microscope, Leica DFC310FX camera [Leica LAS Core software]).

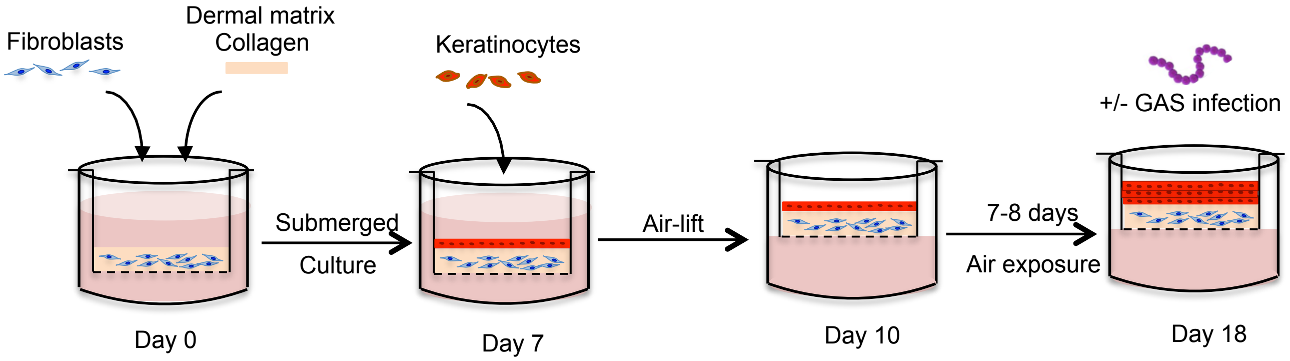

Supplementary Figure 2.

Scheme of the Human 3D organotypic skin model.

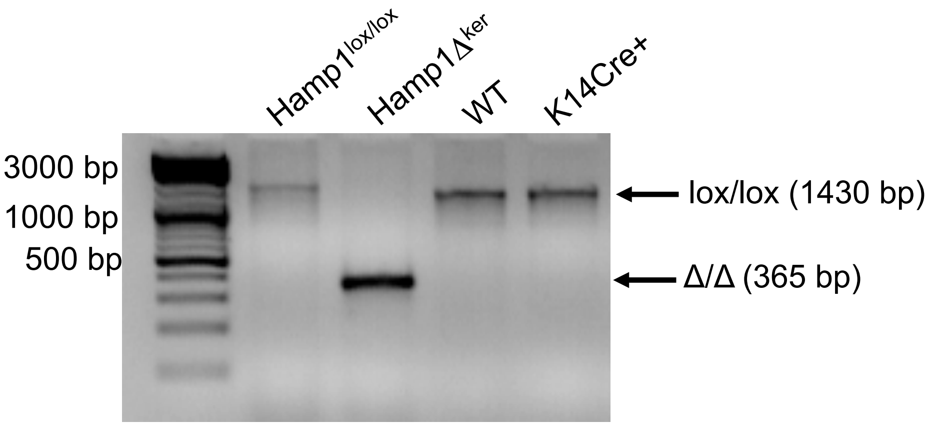

Supplementary Figure 3. Recombination of the floxed hamp1 allele

Genomic PCR in the epidermis of the Hamp1^{lox}/^{lox}, Hamp1Δ^{ker}, WT and K14Cre+ mice.

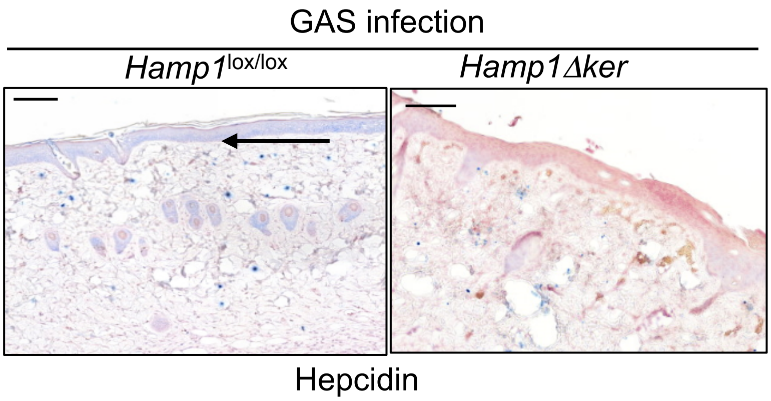

Supplementary Figure 4. Hepcidin (in blue) immunohistochemistry on cutaneous biopsies of Hamp1$^{lox/lox}$ and Hamp1Δ^{ker} mice challenged with GAS for 4 days. Bars represent 100 µm. (5/0.4, Leica DMI3000B microscope, Leica DFC310FX camera [Leica LAS Core software]).

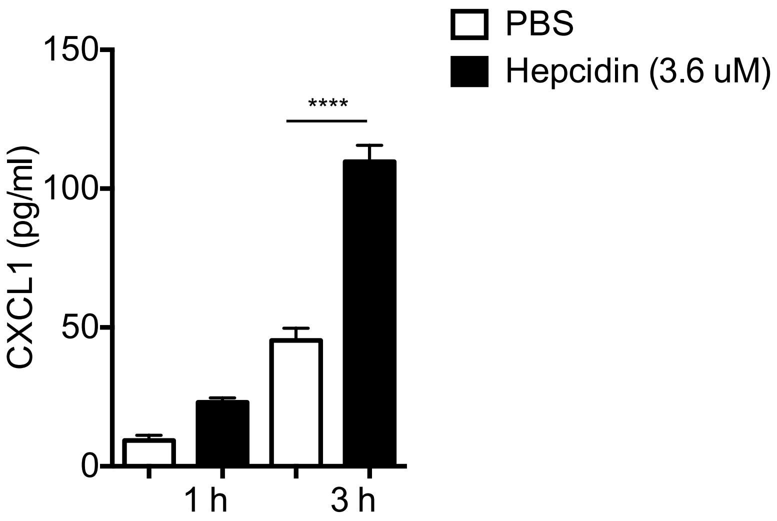

Supplementary figure 5. CXCL1 levels measured by ELISA in the culture supernatant of murine primary keratinocytes stimulated for 1 or 3 hours with hepcidin or PBS. N=3 per group. Representative of 3 independent experiments. Values are shown as mean ± s.e.m. Statistical analysis was performed using a one-way analysis of variance (ANOVA) followed by a post-Tukey test. **** $p<0.0001$.

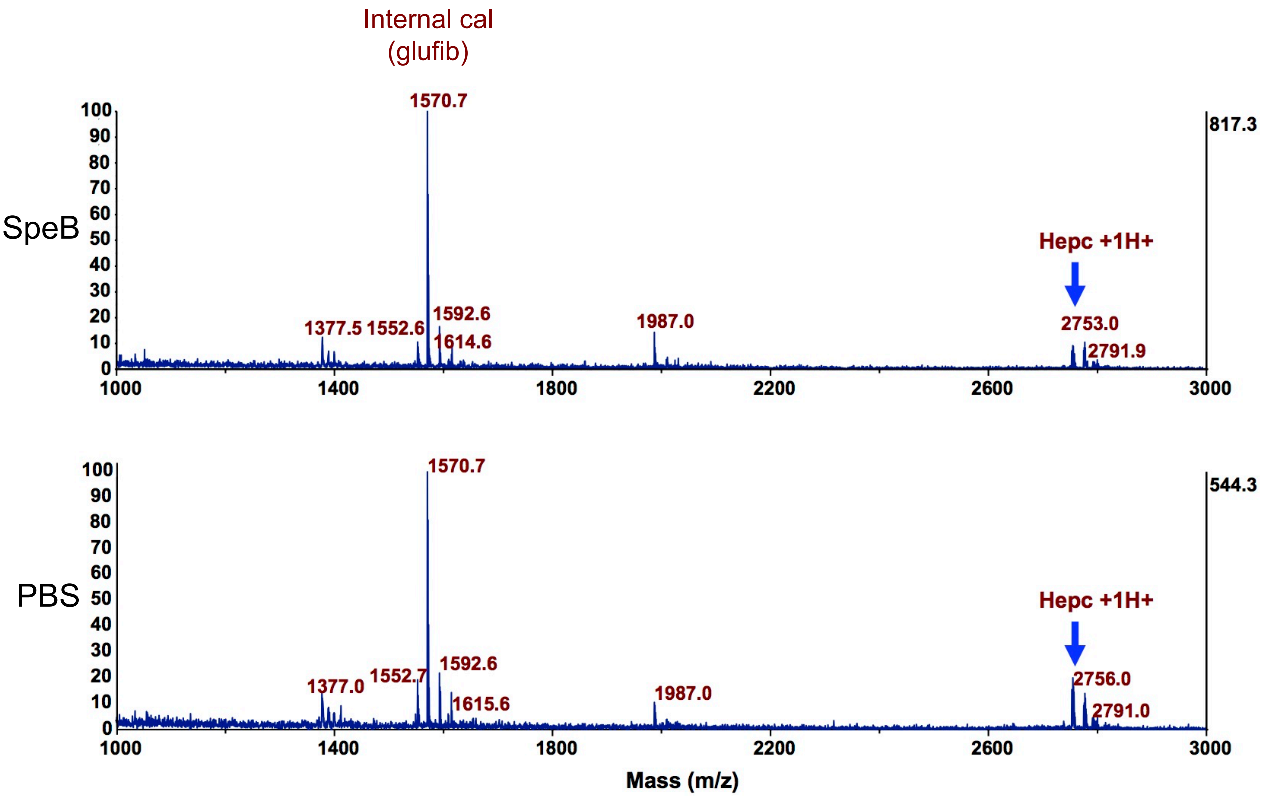

Supplementary Figure 6. Hepcidin is resistant to SpeB cleavage

Mass spectrometry analysis of hepcidin incubated overnight with 2ng SpeB or PBS. Matrix-assisted LASER desorption ionization (MALDI)-TOF MS generated singly (2753 = $M+1H^+$) and doubly (1377 $M+2H^+$) monoisotopic charged ions (indicated as m/z; mass-to-charge ratio) from which the average molecular mass (m) of each was deduced. The blue arrows indicate uncleaved peptide peaks at 2,7 kDa (hepcidin).

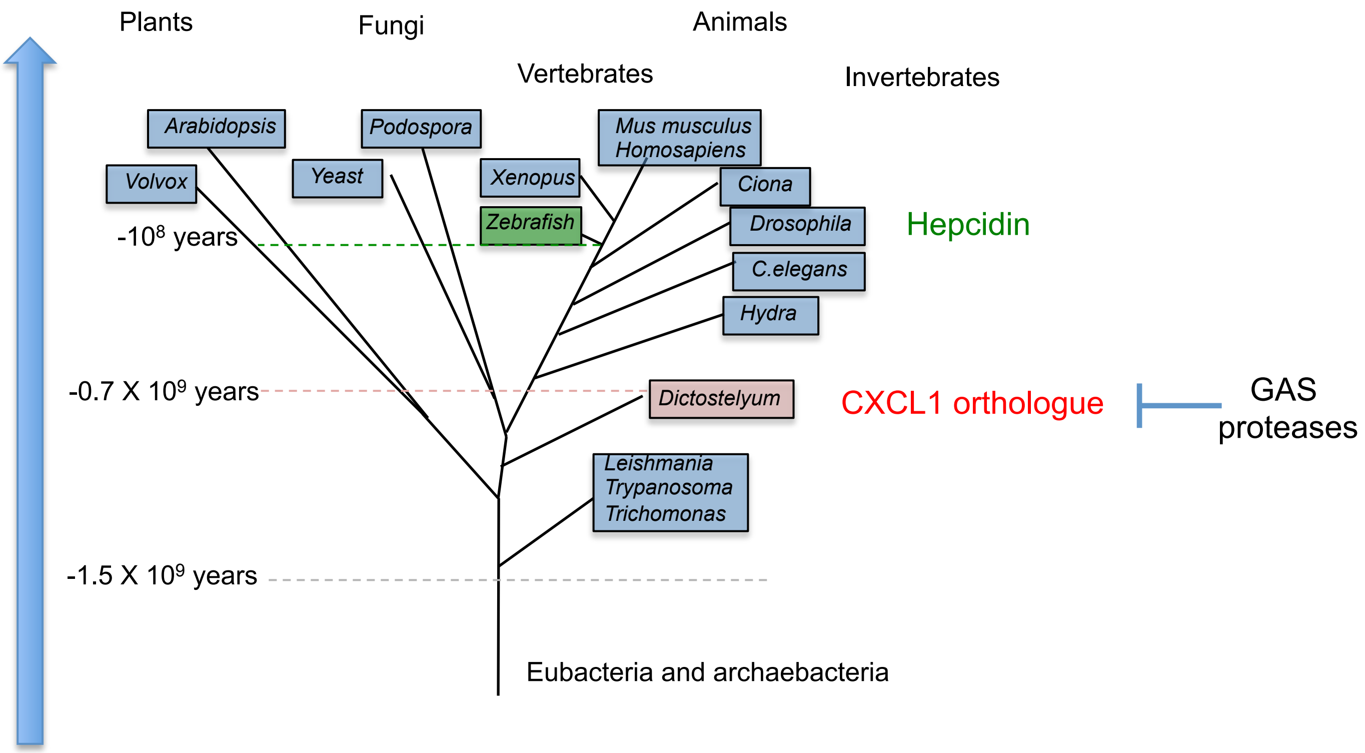

Supplementary Figure 7. Phylogenetic tree showing CXCL1 (in pink) and hepcidin (in green) apparition during evolution. During the co-evolution host-pathogen, GAS has developed a counterattack against CXCL1.

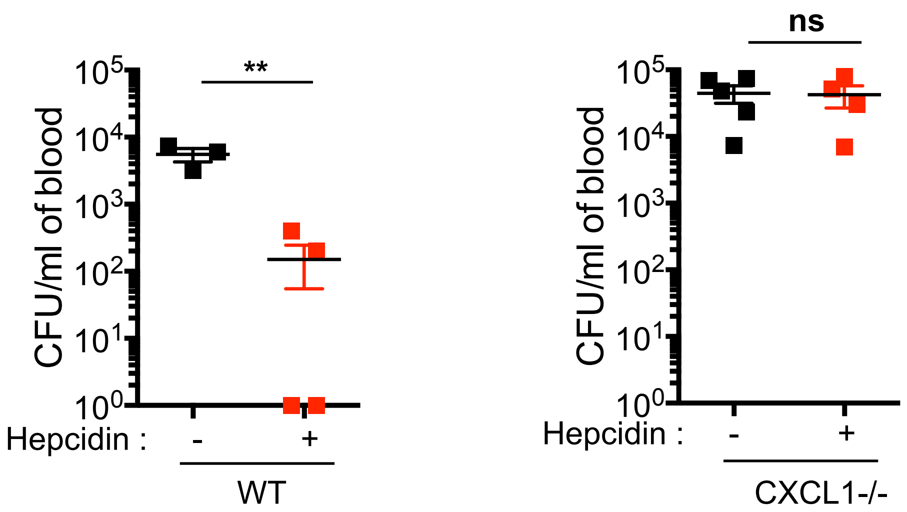

Supplementary Figure 8. Bacterial count in the blood of WT (black square) and CXCL1-/- (red square) infected mice treated with daily subcutaneous injections of hepcidin or PBS during 4 days. Values are shown as mean ± s.e.m. Statistical analysis was performed using unpaired Student's t-test. ** $p<0.01$

Patient No.	Gender	Age (y)	Site	Underlying disease	Preceeding trauma	Type of infection	Sepsis
1	Male	51	Leg	None	Yes	Group A Streptococcus	Yes
2	Female	57	Leg	None	No	Group A Streptococcus	No
3	Female	72	Leg	None	No	Group A Streptococcus	Yes
4	Male	27	Thumb	None	Yes	Group A Streptococcus	No

Supplementary Table 1. Clinical features of NF patients

METHODS

Mice

Hamp1$^{lox/lox}$ mice (1) were bred with K14-Cre transgenic mice (2), in which the Cre recombinase is under the control of the murine K14 promoter. CXCL1-/- mice were kindly provided by Dr Sergio Lira (Icahn School of Medicine at Mount Sinai, New York). Studies were performed in a C57BL6 background, using 8-12 week old male littermates.

Mouse model of GAS infection

A well-established model of necrotizing soft tissue infection was adapted for this study (3, 4). In brief, *Hamp1*$^{lox/lox}$ and *Hamp1*Δ^{ker} mice male littermates were subcutaneously injected with 10^7 c.f.u. of M1T1 5448 GAS. Weight loss and lesion size of mice were estimated daily. After 96 hours, mice were sacrificed and skin necrotic ulcers, spleen, and blood (via retro orbital bleeding) were recovered and homogenized in sterile PBS. Serial dilutions of tissue homogenate were spread on THA plates to enumerate bacterial colony forming units (c.f.u.).

For the rescue experiment, *Hamp1*$^{lox/lox}$ and *Hamp1*Δ^{ker} littermates were subcutaneously infected with 10^7 c.f.u. of GAS with PBS or 1 μg/mouse of recombinant CXCL1 (rCXCL1; R&D system, 1395-KC-025/CF). Mice were intralesionally injected again at 24, 48 and 72 h with PBS or 1 μg of rCXCL1.

For the therapeutic treatment, C57BL6 mice were infected as described above. One day after infection, animals were subcutaneously injected with PBS (control) or 1 μg of hepcidin (Peptide International). Mice were treated again at 48 and 72 hours with 500 ng of hepcidin. After 96 hours, mice were sacrificed and the spleens were collected and homogenized for c.f.u. enumeration.

Immunohistochemistry

Tissues were fixed in 10% formaldehyde and embedded in paraffin. Sections were cut at a thickness of 3 µm and then subjected to pressure cook antigen retrieval and immunohistochemistry (Impact Novared SK48-05 or blue AP SK-5300, Vector) with primary antibodies against keratin 14 (1/1000, PRB-155P Covance), CXCL1 (1/100, NBP1-51188 Novus) and hepcidin (1/100, HEPC11-A Alpha Diagnostic)

A solution of protease K diluted at 1/30 in PBS for 5 min was necessary for unmasking sections before incubating with rat monoclonal anti-PMN primary antibody (1/200, sc-71674 Santa Cruz). Human tissues were incubated overnight at 4°C with a rabbit polyclonal to Hepcidin-25 (1/50, Ab30760 Abcam) and then stained with the ABC-peroxidase technique.

Iron measurement

Plasma and iron levels were quantified colorimetrically by a previously described method (1).

Isolation and treatment of keratinocytes.

Epidermis was obtained from the skin of 2-day-old *Hamp1*$^{lox/lox}$ or *Hamp1*Δ^{ker} mice, as previously described (5). Keratinocytes were isolated by epidermis shaking at 350 rpm for 1 hour at 37°C in stop trypsin medium (DMEM, Thermo fisher 21068028) containing 20% heat inactivated and Chelex (Bio-rad 1421253) treated-FBS, 1% GlutaMAX Supplement, 1% Sodium Pyruvate, 100U/ml Penicillin, 100 mg/ml Streptomycin (Thermo fisher) and 2.5 mg/ml amphotericin B (Gibco). The resulting cell suspension was filtered (100 µm), centrifuged at 250 g for 10 min at 4°C, plated on fibronectin and bovine collagen I coated tissue culture plate and incubated at 37°C in a humidified 5% CO_2 atmosphere. The cells were cultured in 0.07 mM calcium medium (Low Ca^{2+}) containing EGF (10 ng/ml) and cholera toxin (10^{-10}mol/L). Once confluence reached, cells were switched in 1.2 mM calcium medium

(High Ca^{2+}) for ensuring their differentiation. After three days in High Ca^{2+} cells were differentiated and suitable for different experiments.

The human keratinocyte cell line HaCat (CLS, Heidelberg, germany), was cultured in low calcium medium (DMEM, thermo fisher 21068028). Once confluence reached, cells were switched in 2.8 mM calcium medium (High Ca^{2+}) to ensure their differentiation.

Human 3D organotypic skin tissue model

The skin models were generated following previously published protocols (6, 7). The skin model includes human N/TERT-1 cells maintained in EpiLife medium (Invitrogen) and NHDF dermal fibroblasts cultured in DMEM (Invitrogen) supplemented with 10% (v/v) FBS (Invitrogen) cultured at 37°C under a 5% CO_2 atmosphere. Briefly, dermal equivalents were generated by adding NHDF cells (0.4 x 10^5) and collagen (2 mg/ml). After 7 days, 1 x 10^6 N/TERT-1 cells were seeded onto the dermal equivalent. Skin models were then air exposed and incubated for up to 7 d at 37 °C under a 5% CO_2 atmosphere, and the culture medium was replaced every second day in the outer chamber. After air exposure, the models were infected with 0.5 x 10^6 bacteria (GAS) for 24 h. After infection, the tissue culture supernatants were collected for cytokine measurements and skin tissue models were processed for mRNA isolation.

Reverse transcription and real-time quantitative PCR

RNA extraction, reverse transcription and quantitative PCR have been performed as previously described (1). All samples were normalized to the threshold cycle value for cyclophilin-A. The primers used are presented in the table below.

Gene	Forward	Reverse
Cyclophilin A	5'-GTCAACCCCACCGTGTTCTT-3'	5'-CTGCTGTCTTTGGGACCTTGT-3'
Murine Hamp1	5'-CCTATCTCCATCAACAGAT-3'	5'-TGCAACAGATACCACACTG-3'
Human HAMP	5'-CTCTGTTTTCCCACAACAGACG-3'	5'-CGTCTTGCAGCACATCCCACAC-3'

Quantification of cytokines

Keratinocytes, previously starved of FBS for 1 hour, treated or not with 100 uM ferroportin inhibitor 2D-014 (Key Organics) - named compound-1 in (8) - were stimulated with 1 or 10 μg/ml hepcidin (Peptide International) or with 500 μM of Ferric Ammonium Citrate (FAC) and incubated at different time points. The supernatant cytokines were measured with the V-PLEX Proinflammatory Panel1 kit (Meso Scale Discovery) or with ELISA kit (MKC00B, R&D Systems) according to the manufacturer's instruction. HaCat and human 3D organotypic skin model supernatants were analyzed with the V-PLEX Plus Chemokine Panel 1 (human) Kit (K15047-Series, Meso Scale Discovery) or with the Human IL-8/CXCL8 DuoSet ELISA (DY208, R&D System).

Antimicrobial assays

As previously described (9), early log phase GAS were diluted in THB to $\approx 2 \times 10^5$ c.f.u. .ml^{-1}. This bacterial suspension was grown for 16h at 37°C in presence of PBS, LL-37 (Sigma 94261), hepcidin (peptide international) or penicillin G (sigma P7794) and the OD_{600} followed. For antimicrobial killing kinetics, exponential phase GAS was exposed to PBS, LL-37 or hepcidin and incubated at 37°C. At different times point dilutions were plated for the c.f.u. enumeration.

Neutrophil count

Infected skin lesions from *Hamp1*$^{lox/lox}$ and *Hamp1*Δ^{ker} mice were dissociated by shaking at 80 rpm for 90 minutes at 37°C in RPMI containing 5% heat inactivated FBS, 100U/ml Penicillin, 300µg/ml LIBERASE TL and 50 U/ml of DNAse1 mg/ml. The suspension was filtered though a 70 and a 30 µm strainer in PBS 1% FBS and stained with Alexa Fluor® 700 anti-mouse CD45 antibody (Biolegend, 103128) and PE Rat Anti-Mouse Ly-6G (BD Bioscience, 561104). Samples were analyzed with a BD Accuri™ C6 flow cytometer.

Keratinocyte killing assay

Hamp1$^{lox/lox}$ and *Hamp1*Δ^{ker} primary keratinocytes were incubated without antibiotic for 2 hours and then infected with GAS (MOI=10). GAS were centrifuged onto keratinocytes at 350 g for 10 min, allowing bacteria-cell interaction. Infected cells were incubated at 37°C/5% CO_2 for different time points. At each time point, well contents were harvested and spread on THA plates for enumerating c.f.u.

Mass spectrometry and analysis MALDI

Peptide dilutions series were mixed 1:1 on a MALDI target plate with 5mg/ml of alpha-cyano-4-hydroxycinnamic acid matrix (Laser Biolabs) dissolved in of 70%ACN 0.1% TFA 30%milliQ H2O, with 3µM Glu-fibrinopeptide B as internal standard and let to dry on a 96-wells OptiTOF MALDI plate target (ABSciex). Mass spectra were acquired and processed at the 3P5 proteomics facility of the Université Paris Descartes with a 4800 MALDI-TOF/TOF analyser (ABSciex) with a Nd:YAG pulsed LASER (355nm) at 200Hz shooting frequency in positive reflectron mode, fixed fluency, low mass gate and delayed extraction. 500 to 1000

spectra were summed with the 4000series explorer software (ABSciex) by steps of 50 spectra in the range of 1300 to 9000 M/Z (CXCL1) or 500 to 4000 M/Z (HepC). Monoisotopic values were labeled from isotopes clusters, when accessible, with a minimal s/n ratio of 1000. Internal calibration was performed on Glu-fibrinopeptide B at M/Z 1570.68.

Study approval

For human studies, informed consent to the protocol was obtained for all subjects and was approved by the Institutional Review Board and the regional ethics committee Paris IV (IRB 2016/40NICB), n° IRB 00003835. The collection of personal data was approved by the Commission Nationale de l'Informatique et des Libertés (CNIL).

The animal studies described here were reviewed and approved (Agreement n° CEEA34.CP.003.13) by the "Président du Comité d'Ethique pour l'Expérimentation Animale Paris Descartes" and are in accordance with the principles and guidelines established by the European Convention for the Protection of Laboratory Animals (Council of Europe, ETS 123, 1991).

Statistical analysis

Values in the figures are expressed as mean +/- SEM. Analysis was performed using GraphPad Prism 6.0. The significance of experimental differences was evaluated by an unpaired (2-tailed) Student's t-test or a Mann Whitney test, when comparing two groups.

A one-way or a two-way ANOVA followed by a Tukey analysis was used when more than two groups were compared. Statistical significance is indicated by * symbols (*$p < 0.05$, **$p < 0.01$, ***$p < 0.001$, ***$p < 0.0001$). ns indicates not significant.

REFERENCES

1. Zumerle S, Mathieu JR, Delga S, Heinis M, Viatte L, Vaulont S, and Peyssonnaux C. Targeted disruption of hepcidin in the liver recapitulates the hemochromatotic phenotype. *Blood.* 2014;123(23):3646-50.
2. Jonkers J, Meuwissen R, van der Gulden H, Peterse H, van der Valk M, and Berns A. Synergistic tumor suppressor activity of BRCA2 and p53 in a conditional mouse model for breast cancer. *Nat Genet.* 2001;29(4):418-25.
3. Datta V, Myskowski SM, Kwinn LA, Chiem DN, Varki N, Kansal RG, Kotb M, and Nizet V. Mutational analysis of the group A streptococcal operon encoding streptolysin S and its virulence role in invasive infection. *Mol Microbiol.* 2005;56(3):681-95.
4. Peyssonnaux C, Datta V, Cramer T, Doedens A, Theodorakis EA, Gallo RL, Hurtado-Ziola N, Nizet V, and Johnson RS. HIF-1alpha expression regulates the bactericidal capacity of phagocytes. *J Clin Invest.* 2005;115(7):1806-15.
5. Lichti U, Anders J, and Yuspa SH. Isolation and short-term culture of primary keratinocytes, hair follicle populations and dermal cells from newborn mice and keratinocytes from adult mice for in vitro analysis and for grafting to immunodeficient mice. *Nat Protoc.* 2008;3(5):799-810.
6. Siemens N, Chakrakodi B, Shambat SM, Morgan M, Bergsten H, Hyldegaard O, Skrede S, Arnell P, Madsen MB, Johansson L, et al. Biofilm in group A streptococcal necrotizing soft tissue infections. *JCI Insight.* 2016;1(10):e87882.
7. Siemens N, Kittang BR, Chakrakodi B, Oppegaard O, Johansson L, Bruun T, Mylvaganam H, Group IS, Svensson M, Skrede S, et al. Increased cytotoxicity and streptolysin O activity in group G streptococcal strains causing invasive tissue infections. *Sci Rep.* 2015;5:16945.
8. Ross SL, Biswas K, Rottman J, Allen JR, Long J, Miranda LP, Winters A, and Arvedson TL. Identification of Antibody and Small Molecule Antagonists of Ferroportin-Hepcidin Interaction. *Front Pharmacol.* 2017;8:838.
9. Nizet V, Ohtake T, Lauth X, Trowbridge J, Rudisill J, Dorschner RA, Pestonjamasp V, Piraino J, Huttner K, and Gallo RL. Innate antimicrobial peptide protects the skin from invasive bacterial infection. *Nature.* 2001;414(6862):454-7.

Uncut gel of Supplementary Figure 3

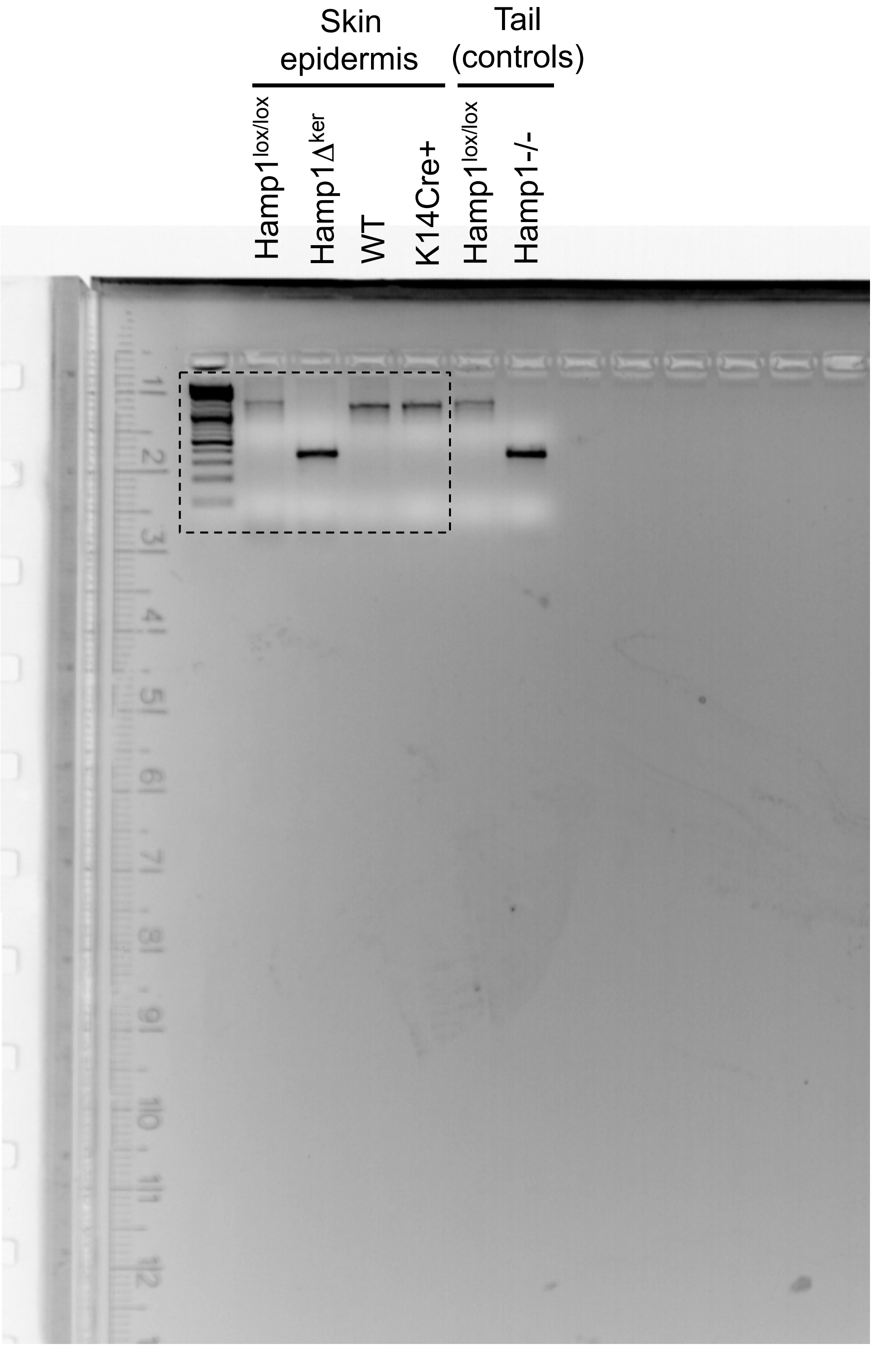

Supplement: Supplemental data [file jci-130-126645-s189.pdf]
